# Chronic cement dust load induce novel damages in foliage and buds of *Malus domestica*

**DOI:** 10.1038/s41598-020-68902-6

**Published:** 2020-07-22

**Authors:** Kamran Shah, Na An, Wenchun Ma, Gulshan Ara, Kawsar Ali, Svetlana Kamanova, Xiya Zuo, Mingyu Han, Xiaolin Ren, Libo Xing

**Affiliations:** 1 College of Horticulture, Northwest Agriculture and Forestry University, Yangling, 712100 Shaanxi China; 2 Institute of Biotechnology and Genetic Engineering, The University of Agriculture, Peshawar, Pakistan; 3 Agronomy Department, Abdul Wali Khan University Mardan, Mardan, Pakistan; 4 College of Food Science, Northwest Agriculture and Forestry University, Yangling, 712100 Shaanxi China

**Keywords:** Plant sciences, Environmental sciences

## Abstract

Cement industry-derived pollutants appear to play multiple roles in stimulating abiotic stress responses in plants. Cement dust deposition on agriculture fields can affect soils, photosynthesis, transpiration and respiration of plants. Here, we characterised the acute physiological responses of Malus × domestica leaves to different cement dust concentrations. The cement dust was sprinkled over plants daily for 2 months at 10 and 20 g/plant, with 0 g/plant serving as the control. Leaf physiological responses revealed significant increases in oxidative stress and antioxidant enzyme activity levels. Additionally, ascorbic acid, soluble sugar, free amino acid, and pigment levels decreased after exposure to cement dust. Macroscopic morphometric parameters, such as weight, dry matter content, and lengths and widths of leaves and buds, were significantly reduced in the cement-treated groups. A histological analysis of leaves and buds revealed decreased cellular areas, cellular damage, and abridged leaf thickness, while an ion leakage assay confirmed the negative effects on tissue integrity. These results provide evidence that cement dust is a hazardous pollutant that induces abiotic stress responses and has degradative effects on leaf health, pigment and biochemical metabolite levels, and anatomical features. Studies to determine the elemental residues of cement dust present in edible plant parts and the adverse impacts of their consumption on human health are strongly recommended.

## Introduction

Anthropogenic processes involved in rapid industrialisation and mechanisation are polluting the earth^1^ by releasing significant amounts of pollutants into the atmosphere. Harmful gases and other wastes, such as dust and heavy metals, are also emitted into the atmosphere regularly, resulting in substantial negative effects on human health, plant development, agriculture output, and natural ecosystems^2,3^. The addition of toxic pollutants into the atmosphere at the global level has contributed to climate change and increased the severity of stresses affecting living organisms^4^. For example, poor air quality is harmful to plants, because they directly interact with the atmosphere and are, generally, immobile^5^. The poor air quality associated with the cement manufacturing industry is receiving attention owing to its increased concentration of particulate matter, which causes major damage to leaves and hinders the growth and development of horticultural plants^6,7^.

Cement manufacturing processes generate a tremendous amount of dust particulates (coarse and fine), which decrease the surrounding air quality. Their emission of considerable amounts of toxic gases and particulate matters into the atmosphere is causing significant air pollution^8^. Cement contains oxides of sulphur and nitrogen, which damage vegetation by affecting their gas exchange processes^9,10^. Cement dust deposits on plants interfere with the biosynthesis of chlorophyll and damage leaf cells, resulting in a reduction in photosynthesis^11–13^. In some cases, the dust deposition stiffens/weakens flower buds, resulting in bud drop^14^. Oxides of cement dust may react with water droplets, forming acid rain, which damages the soil vegetation, and land^15^. Heavy metals, such as chromium, nickel, cobalt, lead, and mercury, are readily found in cement dust^16^. The release of cement dust particles, along with heavy metals, has created severe environmental and health issues^17,18^. Their harmful effects extend to human health, causing skin irritation and damaging the mucous membrane of the eyes as well as the respiratory system^19^.

Cement pollutants may passively enter plant leaves during gas-exchange processes^20^ and then enter the intercellular spaces of plant tissues, interrupting the reaction centres of plants to impair metabolism^21,22^. Cement dust incorporated in phytotoxic gas pollutants in fruit plants weaken respiratory, photosynthetic, and transpiration-related processes^21,23^. The emission of particulate matter, such as roadside dust from unpaved and dusty roads^6,24,25^ and cement dust from cement industries^26,27^, contributes to the coating of surrounding habitats and vegetation^28^, resulting in stunted plant growth and reduced production^29^. Cement dust-induced toxicity is an emerging environmental and horticultural problem that limits fruit plant productivity and also makes the fruit harmful for human and animal consumption^30^.

*Malus* × *domestica* (*MD*) is an economically and culturally significant fruit plant^31,32^ grown worldwide, and its consumption is highly recommended for a healthy human life^33^ because of its useful nutrients, which aid in reducing the risks of heart disease, stroke, and lung cancer^32,34^. Abiotic stresses are environmental issues that directly affect leaf physiology and strongly limit fruit-plant growth and yield worldwide^35–37^. Currently, apple tree farming is challenged by severe pollution problems owing to rapid industrialisation, which has resulted in decreasing plant productivity and performance^38,39^. However, the contribution of cement dust to the degradation of leaf pigments, histological features, and biochemical parameters is unknown in *MD*. Cement industry-derived airborne pollutants pose a threat to plant growth and development because they can travel long distances and deposit on plant leaves as well as soil. Chemical reactions of soil with cement ingredients have resulted in drastic changes in soil chemistry that directly affect plant nutrition, leaf chemistry, and plant growth^40,41^. Thus, cement dust has adverse effects on soil physico-chemical properties and biological activities^42,43^. Cement dust deposits on soil increase the heavy metal levels^44^, pH^45^, electrical conductivity, and bulk density of the soil, while decreasing the water-holding capacity, as well as the moisture, organic carbon, and total nitrogen contents of the soil^46^. Therefore, plants near cement factories suffer from chronic stress owing to the release of cement kiln pollutants, which affect soil chemistry^47^. We investigated the responses of *MD* shoots to chronic exposure to cement dust while the soil was mulched. Cement dust is thought to be the most toxic environmental factor that physically blocks stomata and affects plant growth and development^6,24,25,48^. However, little is known about the effects of cement dust depositions on the morphological, physiological, and histological characteristics of *MD* leaves. We hypothesised that cross-talk responses to cement dust exposure will result in attenuated leaves and buds, leading to their dysfunction, which impedes the healthy growth and production of fruit plants. Therefore, for the first time, a study was designed to investigate the negative effects of cement dust on the physiological and histological parameters of *MD leaves.*

## Materials and methods

### Site description and climatic condition

The experiment was conducted at Fruit garden, Northwest Agriculture and Forestry University, Yangling (34°16′N, 108°04′E), 462 m above sea level. Summer is hot, humid, wet, and partly cloudy and the winter is very cold and dry. The area is semiarid with an annual mean precipitation of 580 mm per year, however a mean annual temperature of 12.9 °C (minimum − 17.4 °C, maximum 42 °C)^49,50^. The soil condition at the experiment site was light silt loam, containing 13.62 g/kg organic carbon, 0.95 g/kg total nitrogen, 137.1 mg/kg potassium, and 22.3 mg/kg available phosphorus at the top 0–40 cm of the soil layer. A temperature ranging from 23.0 to 32.0 °C (night-day) and a photosynthetic photon flux density of approximately 1,500 μmol/m^2^s at midday was the experimental conditions.

### Plants treatment with cement dust and sampling

The experiment was conducted in field and 30 uniform *MD* apple plants in Fruit garden (apple farming field) were selected and divided into three groups (T_0_, T_1_ and T_2_). The distance between 2 plants was about 3.5 m. The experiment was laid out as a completely randomized design, with ten repetitions of each treatment. Cement was sprinkled in air 0.5 m above the plant by using a hand pump daily for a period of 2 months. The experimental plants were grown in rows, and the soil was covered with 1.5 m wide plastic mulches on both sides of the plants to minimize or stop the contact of cement dust with soil. We recorded the soil physiochemical data before and after the experiment and there were no major changes in soil composition upon treating plants with cement dust. Fertilization and irrigation were supplied to each tree by the drip irrigation system. Cement treatments were T_0_ (0 g/plant), T_1_ (10 g/plant), T_2_ (20 g/plant). T_0_ was controlled and maintained in a cement-dust free environment. On a rainy day, we did not sprinkle cement over plants, which were 3 days in the whole period. After 2 months of the experimental period, samples of leaves were collected at mid-day and immediately frozen in liquid nitrogen for biochemical analysis, while for leaf and buds histology; fresh samples were fixed in FAA.

### Morphometric studies

For morphometric analysis, fresh samples of leaves and buds were harvested and brought to the Laboratory of Horticulture. Length and width per 10 buds and 10 leaves were determined by measuring tape. Leaf area (cm^2^) was recorded using portable leaf area meter (LI-COR Inc., Lincoln, NE, USA), while the weight of buds and leaves was determined with Ohaus digital balance (Ohaus Scale Corp. Florham Park, NJ, USA). For the determination of dry matter and water content, we placed leaves and buds in oven for 24 h at 60 °C. Fresh weight and dry weight were recorded to calculate the dry matter and water contents of leaves and buds.

### Analysis of stress markers

Lyophilized samples of leaves were grinded to a fine powder and subjected to analysis of stress markers^51^.

### Determination of Hydrogen peroxide (H_2_O_2_) content

H_2_O_2_ levels were determined according to the previous protocol^52,53^. A total of 500 mg of leaf tissues were homogenized, and 390 nm absorbance of the supernatant was read by spectrophotometer (UV-1201 Shimadzu spectrophotometer, Japan). The concentration was calculated from a standard y curve.

### Determination of Malondialdehyde (MDA) content

MDA was estimated to determine lipid peroxidation in leaves by thiobarbituric acid (TBA)^51^.

### Measurement of enzymatic antioxidants (SOD, CAT, and POD)

For determination of enzymatic antioxidants, we grinded 500 mg leaves, homogenized with 5 mL potassium phosphatase buffer (10 mM, pH 7.0) plus polyvinylpyrrolidone (4% w/v) and centrifuged (Sorvall ST16R, Thermo, USA) at 12,000 × *g* for 30 min at 4 °C. The Supernatant was used to determine catalase (CAT)^54^, peroxidase (POX)^55^, and superoxide dismutase (SD)^56^. POX activity was calculated by using a spectrophotometer (UV-1201 Shimadzu spectrophotometer, Japan) to observe an increase in absorbance at 470 nm in phosphate buffer containing H_2_O_2_ (0.5 mM) and guaiacol (1 mM). One unit of POX is the amount of enzyme that increased the absorbance of 0.01/min. CAT activity was calculated by observing a decrease in absorbance at 240 nm in phosphate buffer (50 mM, pH 7.5) containing H_2_O_2_ (20 mM). One unit of CAT is the amount of enzyme that used 1 mM H_2_O_2_/min. One unit of SOD is the amount which reduces the absorbance reading to 50% compared to control (lack enzyme).

### Measurement of non-enzymatic antioxidants (ascorbic acid)

Ascorbic acid concentration of leaf was determined by following the previous protocol^57^. Briefly, leaf tissue was homogenized with 1.5% (w/v) metaphosphoric acid containing 1 mM ethylenediaminetetraacetic acid, and the extract was subjected to HPLC analysis using water/methanol (3/1, v/v), containing 0.05% (w/v) sodium dihydrogen phosphate monohydrate (pH 3.6) and 1 mM hexadecylammoniumbromide at a flow rate of 1 mL/min for 20 min and photo-detection at 248 nm.

### Determination of free amino acid and total sugar

Lyophilized samples of leaves were analyzed to determine concentration of free amino acid^58^ and total sugar^59^.

### Determination of photosynthetic pigments

Leaf pigments were extracted by using acetone reagent, and extraction procedures were performed in dim light as reported in our previous reports^6,24,25,48^.

### Electrolyte leakage (EL)

For electrolyte leakage, fresh samples of leaves and buds were harvested and brought to the Laboratory of Horticulture. To observe the leaf membrane damage, we follow the previously reported procedure^60^ with minor modification. Fresh leaf samples (0.5 g) were washed with deionized water, placed in tubes containing 10 mL of deionized water followed by incubation for 2 h at 25 °C. Then, the electrical conductivity of the solution (L_1_) was recorded. The samples were then autoclaved for another 20 min at 120 °C and after equilibration at 25 °C, the final conductivity (L_2_) was measured.

### Histology and microscopy

Histological analyses of leaves and buds were executed as previously described^61^ with little changes. Leaf and buds samples were dehydrated with fractionated ethanol series, cleared in dimethyl benzene and transverse sections of leaf blades and buds were collected using a microtome (Leica RM2016) and stained in toluidine blue (EMD Millipore Corporation, catalog number: 1159300025). Bright-field images were taken by Olympus (BX53) microscope. Leaf and bud thickness were determined by ImageJ, https://imagej.nih.gov/ij/, and each aggregate was analyzed in multiple consecutive sections using straight tool of ImageJ and averages were calculated. Cell surface area was measured using freehand selection tool of ImageJ.

### Statistical analysis of data

Graphs were plotted and statistically evaluated using GraphPad Prism version 7.00 for Windows (One-way ANOVA followed by Dunnett test) GraphPad Software, San Diego California USA, www.graphpad.com. *p* < 0.05 was considered statistically significant. Results presented as mean ± SEM. *p < 0.05, **p < 0.01, ***p < 0.001, ****p < 0.0001 and ns = *p* > 0.05.

## Results

### Cement duct induces abiotic stress in *MD*

**Figure 1:**
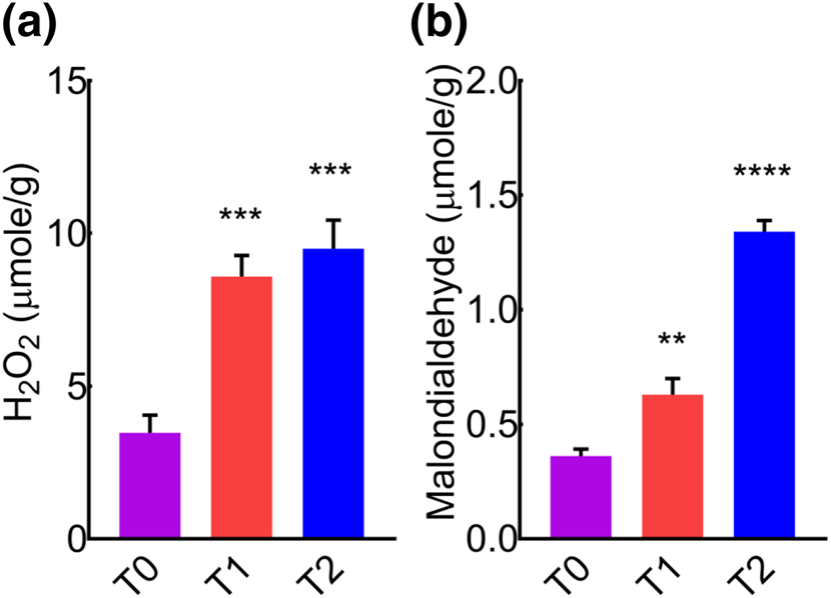
Quantification of leaf malondialdehyde and hydrogen peroxide to check the onset of abiotic stress in *MD* exposed to different concentrations of cement dust. (**a**) Leaf H_2_O_2_ contents. (**b**) Leaf malondialdehyde contents.

Interest in the roles of different abiotic stress factors on plant health has promoted research in the field of horticulture and food sciences. To investigate the induction of stress by cement deposition, we analyzed the level of different reactive oxygen species (ROS) in the leaves of *MD*, as the overproduction of ROS in plants under stress conditions is a common phenomenon^62^. For this purpose, whole leaves extracts were analyzed to determine H_2_O_2_ and MDA concentrations. We found a significant increase in the concentration of H_2_O_2_ and MDA (Fig. 1a,b), that revealed a positive association between cement dust and ROS production. It suggested that cement dust could be estimated as a key abiotic environmental stress factor that could enhance the reactive species and promote the onset of abiotic stress in plants. Next, we presumed that cement induced upsurge of leaf H_2_O_2_ and MDA contents might affect leaves antioxidants activities.

### Enzymatic antioxidant activities increase with cement treatment

**Figure 2:**
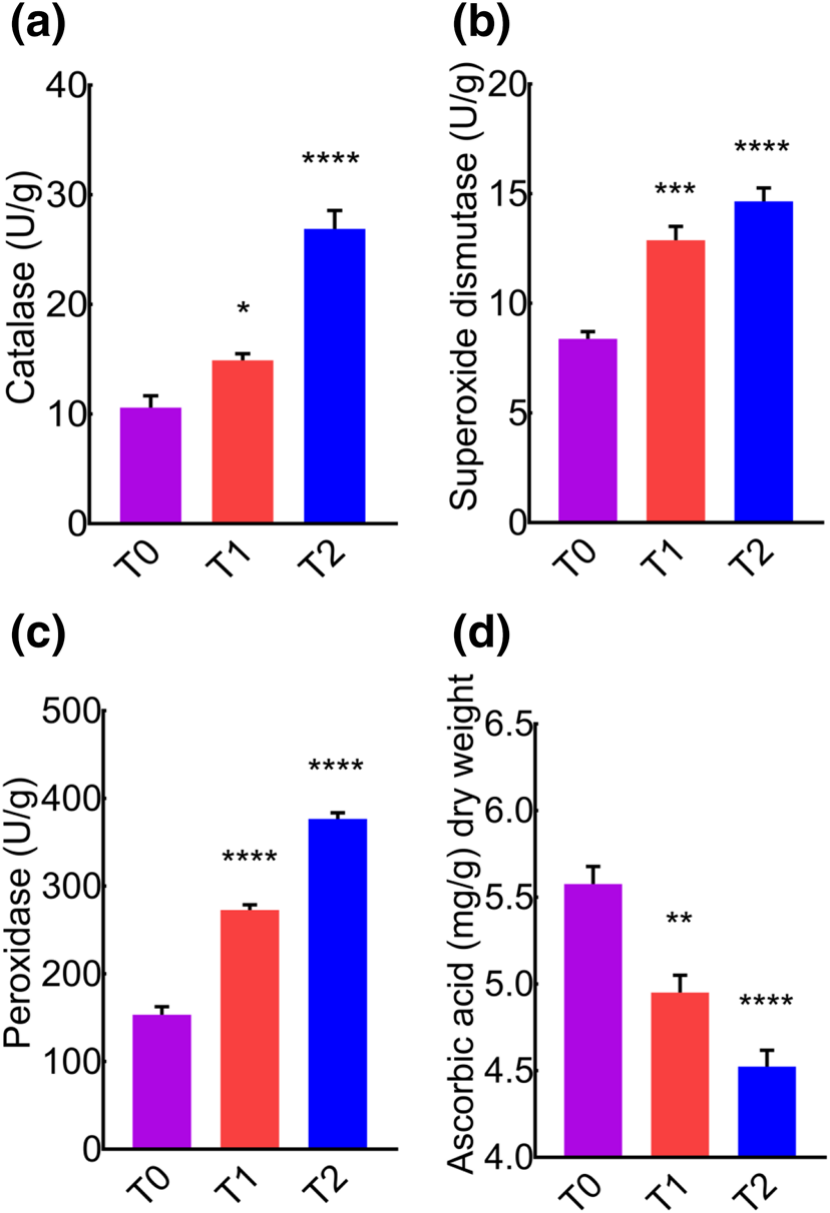
Quantification of enzymatic antioxidants (CAT, SOD, and POX) and non-enzymatic antioxidants (ascorbic acid) in the leaves of *MD* exposed to different concentrations of cement dust. (**a**) Leaf catalase contents. (**b**) leaf superoxide dismutase contents. (**c**) Leaf peroxidase contents. (**d**) Leaf ascorbic acid contents.

The response of enzymatic antioxidants is a key process to defend plants from the damages associated with a variety of environmental stress factors. It is well documented that antioxidant enzymes such as SOD, CAT or POX increase in stress conditions^62^. Here we found a significant increase in the levels of CAT, SOD and POX (Fig. 2a–c) in cement treated groups compared to control (T_0_).

### Non-enzymatic antioxidant activities decrease with cement treatment

The balance between non-enzymatic antioxidants and ROS is essential for maintaining healthy plants. We continued to investigate whether cement also influences the non-enzymatic anti-oxidants. Ascorbic acid is thought to play a vital role in the adaptation of plants to environmental stresses, which regulates the cascades of spontaneous oxidation and prevent plant cells from stress-related damage by hunting of scavenging ROS^63^. Strikingly, we found a significant decrease in the level of ascorbic acid in cement treated groups compared to control (Fig. 2d). These data showed that cement enhances the enzymatic antioxidant (Fig. 2a–c) but have an adverse effect on ascorbic acid (Fig. 2d).

### Variation in biochemical health markers

**Figure 3:**
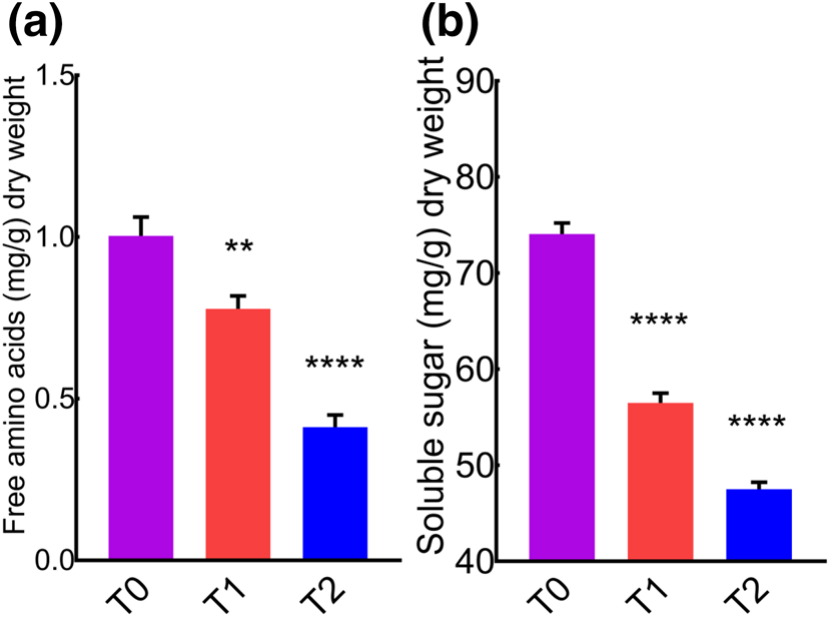
Cement pollution reduces concentrations of free amino acid and soluble sugars in the leaves of *MD*. (**a**) Leaf free amino acid content. (**b**) Leaf soluble sugar content.

Ascorbic acid is required for the normal functioning of living cells. It has been proposed that low ascorbic acid concentration in plants have less stress tolerance due to altered metabolism^64^. Based on this hypothesis, we quantified different biochemical health markers in the leaves of *MD*. We observed a robust decrease in the leaf free amino acids (Fig. 3a) and soluble sugar (Fig. 3b). This suggested that cement dust is a phytotoxic factor of our environment, and their noxious effects in plants are extensive. Taken together, our data suggested that cement also affect leave biochemical constituents.

### Influence of cement on photosynthetic pigments of leave

**Figure 4:**
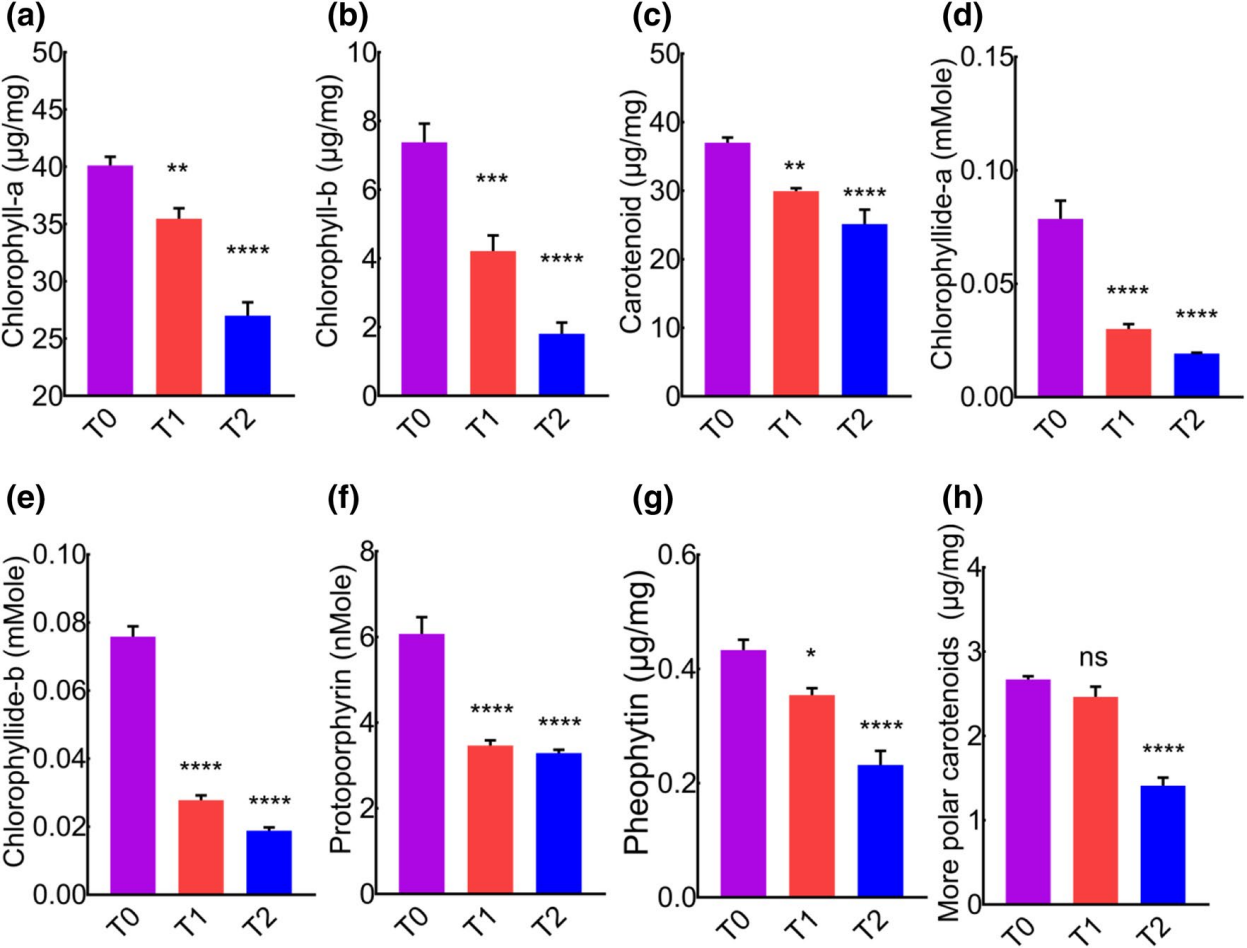
Cement dust deposition reduces different leaf pigments. (**a**) Leaf chlorophyll-a contents. (**b**) Leaf chlorophyll-b contents. (**c**) Leaf carotenoid contents. (**d**) Leaf chlorophyllide-a contents (**e**) leaf chlorophyllide-b contents. (**f**) Leaf protoporphyrin contents. (**g**) Leaf pheophytin contents (**h**) Leaf more polar carotenoid contents.

The inhibition of photosynthesis is a key pathological feature of abiotic stress. Chloroplast damage is the primary consequence of stress in plants, which indicates the degradation of leaf pigments. We next analyzed different leaf pigments in the leaves of *MD*. Patterns of pigment expression were different in control and cement treated plants, where cement dust drastically reduced various leaf pigments. Compared to control, cement treated groups showed less concentration of chlorophyll-a (Fig. 4a), chlorophyll-b (Fig. 4b), carotenoid (Fig. 4c), chlorophyllide-a (Fig. 4d), chlorophyllide-b (Fig. 4e), protoporphyrin (Fig. 4f), pheophytin (Fig. 4g), and more polar carotenoid (Fig. 4h). It indicated that the biosynthesis of leaf pigments are interlinked with each other, and effect on one pigment could influence other pigments, and hence overall pigment degradation happened. Our data suggested that cement notorious effects on leaf pigments are extensive that might influence plant performance and morphometric properties.

### Morphometric studies

**Figure 5:**
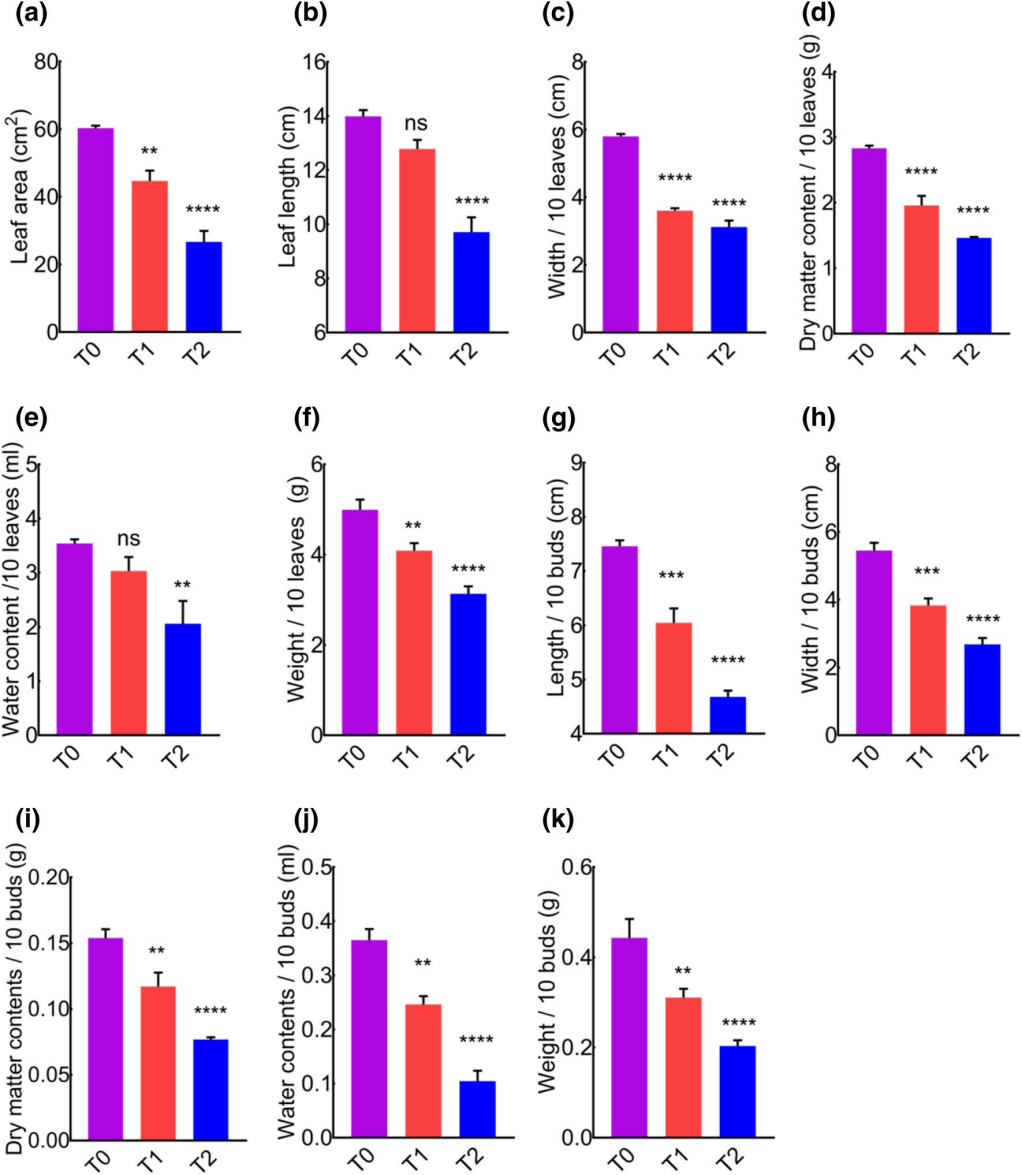
*MD* morphometric features were affected by different concentrations of cement deposition. (**a**) Average leaf area. (**b**) Average leaf length. (**c**) Average width per 10 leaves. (**d**) Average dry matter contents per 10 leaves. (**e**) Average water contents per 10 leaves. (**f**) Average weight/10 leaves. (**g**) Average length per 10 buds. (**h**) Average width per 10 buds. (**i**) Average dry matter contents per 10 buds. (**j**) Average water contents per 10 buds. (**k**) Average weight per 10 buds.

Abiotic stress enhances the production of ROS and causes pigments degradation, which might also cause cell death, and morphometric properties could be affected. Next, we sought to determine the influence of cement on leaves and buds macroscopic-morphometric properties. Area, length and width/10 leaves were significantly reduced by cement dust (Fig. 5a–c). Moreover, the dry matter content, water contents and weight/10 leaves were significantly reduced in cement treated groups (Fig. 5d–f). Similarly, the length and width/10 buds were found shorter in cement treated groups (Fig. 5g–h). Furthermore, dry matter content, water content and weight/10 buds were significantly reduced in cement treated groups compared to control (Fig. 5i–k). This reduction in morphometric parameters could be attributed to the negative impacts of cement dust on plant performance.

### Histological examination of leaves and buds

**Figure 6:**
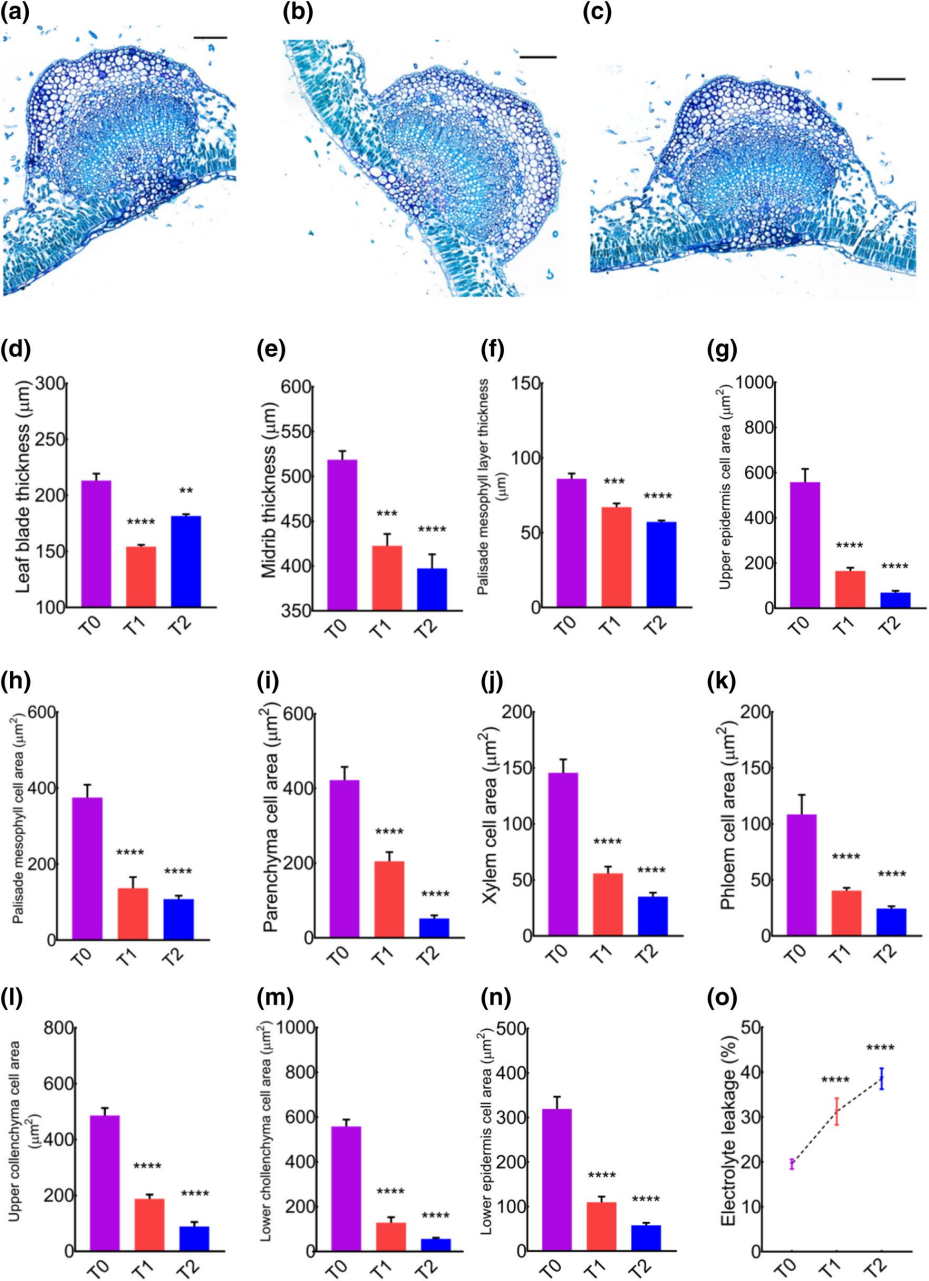
Microscopic analysis of leaf transverse sections revealed adverse effects on the morphometric properties of tissues in cement treated groups. (**a**–**c**) Detailed micrographs of transverse sections of the *MD* leaf blade of T_0_, T_1,_ and T_2_ groups. (**d**–**f**) Average leaf blade-, midrib-, and palisade mesophyll layer-thickness in μm. (**g**–**n**) Average upper epidermal-, palisade mesophyll-, parenchyma-, xylem-, phloem-, upper collenchyma-, lower collenchyma-, and lower epidermis-cell areas in μm^2^. (**o**) Percent of electrolyte leakage in leaves of control and cement treated groups.

Leaves are the main source of photosynthesis and are highly exposed to environmental stresses. To elucidate the effects of cement induced stress on cell-based phenotypic characteristics, histological analysis of leaves and buds were performed to reveal micro-phenotype at the cellular and tissue level. Leaf-blade transverse sections of T_0_, T_1_ and T_2_ are shown in Fig. 6a–c. Cells were less dense and less compact in histological sections of cement treatment groups. Furthermore, leaf blade thickness, midrib thickness, palisade mesophyll layer thickness was significantly reduced in cement treated groups (Fig. 6d–f). We measure cell surface areas of different cells by using ImageJ. Upper epidermis cell-, palisade mesophyll cell-, parenchyma cell-, xylem cell-, phloem cell-, upper collenchyma cell-, lower collenchyma cell-, and lower epidermis cell-areas were significantly reduced in T_1_ and T_2_ groups compared to control (Fig. 6g–n), which signposted the toxic effects of cement dust on cross-sectional areas of different leaf cells. We counter confirmed the damage caused by cement dust by performing electrolyte leakage in leaves and found a robust increase in ion leakage in leaves of cement-treated groups (Fig. 6o). An increase in ion leakage in cement treated groups provides confirmatory evidence of leaf damage exposed to cement dust.

**Figure 7:**
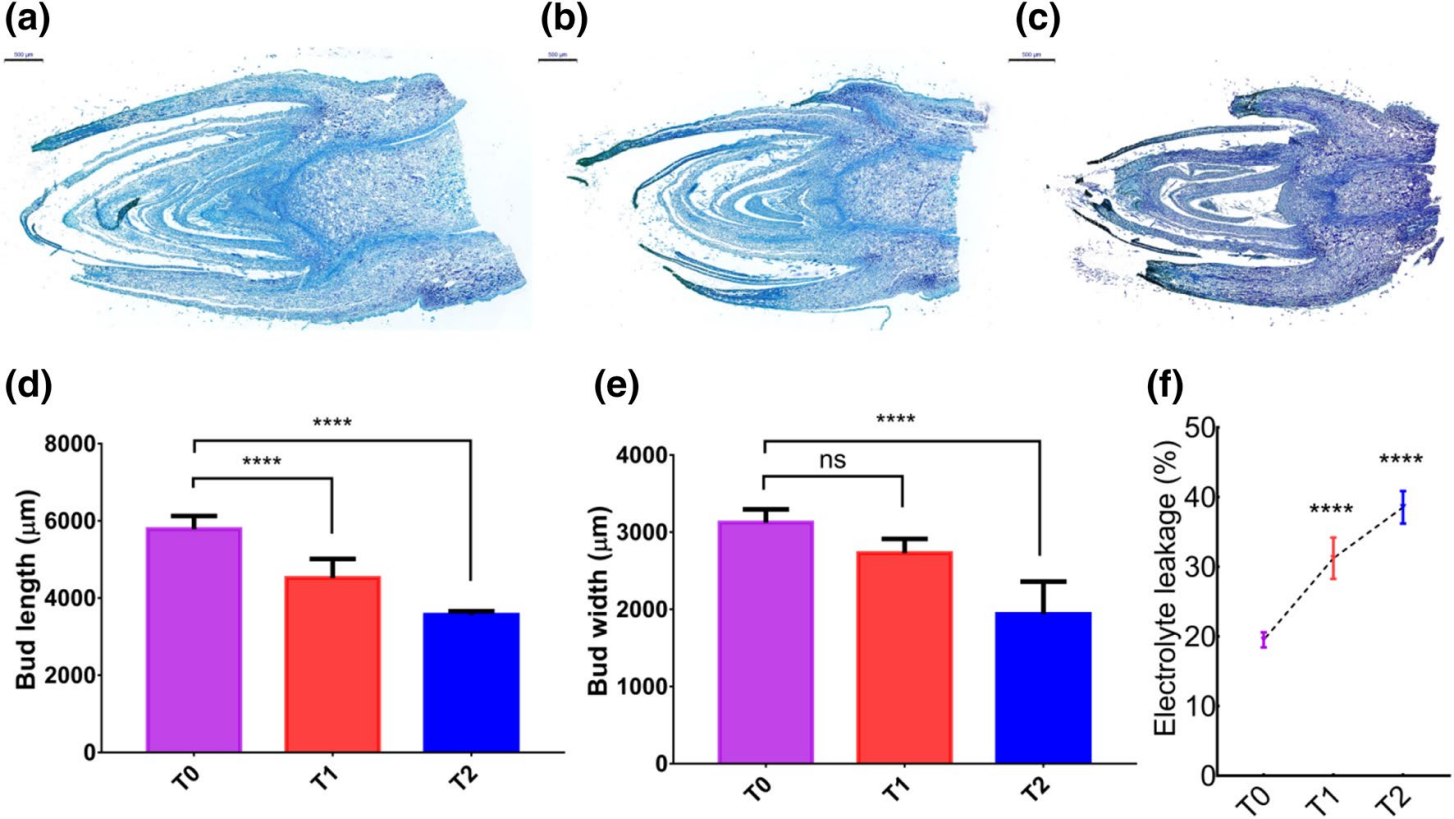
Microscopic analysis of buds transverse sections revealed adverse effects on phenotypic properties of buds in cement treated groups. (**a**–**c**) Detailed micrographs of transverse sections of *MD* bud of the T_0_, T_1,_ and T_2_ groups. (**d**–**e**) Average bud length and width in μm. (**f**) Percent of electrolyte leakage in buds of control and cement treated groups.

**Figure 8:**
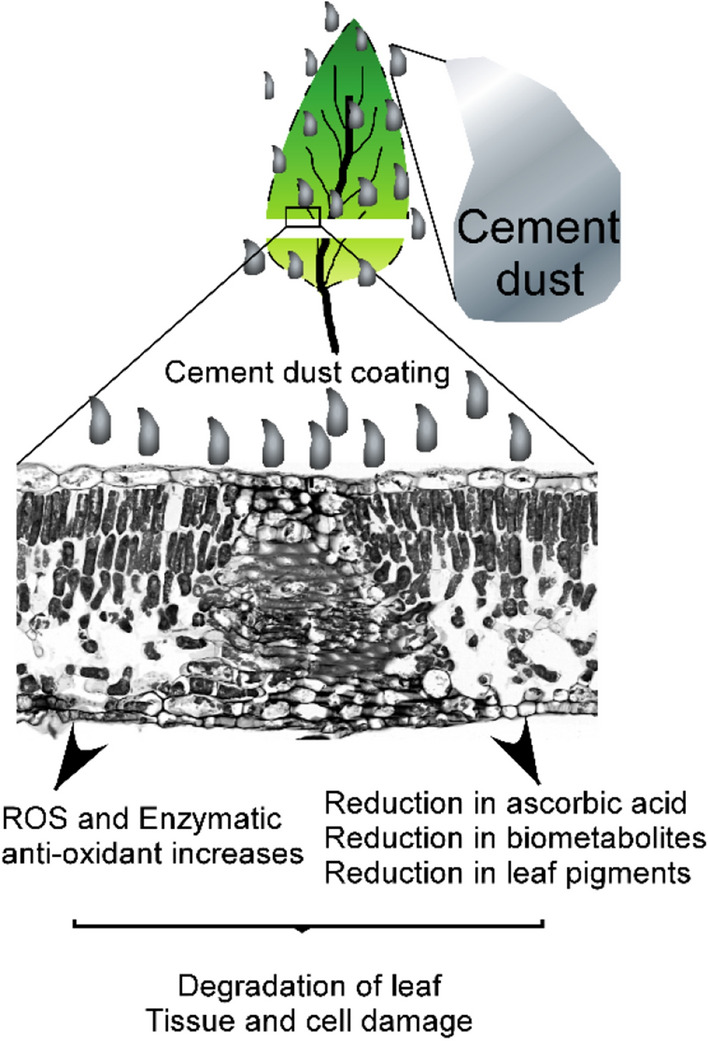
Schematic illustration of cement dust deposition on leaves of *MD* plant.

We noticed the reduction in width, length and weight of buds in cement treated groups compared to control (Figure S1). Next, we analyzed buds histology to uncover the phenotypic changes at the cellular/tissue level. Figure 7a–c showed transverse sections of buds. Measurement of buds length (Fig. 7d) from the histological section shows a significant reduction of bud length in cement treated groups. In the case of bud width (Fig. 7e) we observed no significant difference between control and T1; however, a significant reduction in T2 treatment compared to control. Further, we analyzed the ion leakage in buds tissues to check cell damage. Strikingly, the ion leakages of cement-treated groups were significantly higher than control (Fig. 7f), which confirmed that cement-treated plants were experiencing more membrane injuries and cell damage. Cement dust is estimated as a risk factor for plant growth and development. In summary, our data showed that cement dust load induces abiotic stress and has degradative effects on leaf histology. The cement dust deposition increases the leaves reactive oxygen species and enzymatic antioxidants while reduces the leaves ascorbic acid, biometabolites, and pigments (Fig. 8).

Chronic dust deposition increases leave reactive oxygen species and enzymatic antioxidants, while reduces leaves ascorbic acid, biometabolites, and pigments, resulting in novel damages to leaves tissues.

## Discussion

During the last decade, cement production, and, consequently, the emission of cement dust, have risen owing to the increased demand for cement materials used in construction. Anthropogenic activities that cause environmental changes are expected to exacerbate the poor air quality in different regions of the world. We found that chronic exposure to cement dust induced novel damage in *MD* leaves. This may be caused by the heavy metals and other elements present in cement dust, which affect the foliar surfaces of leaves. These undesirable effluents changed cellular morphology and modulated the biochemical constituent and pigment contents of leaves, resulting in massive damage owing to the persistent presence of the pollutants. This indicated that cement dust may cause significant injuries to a variety of fruit plants. The persistent hostile environment worsens the effects, making them more extensive.

Plants adjust their metabolic activities in response to environmental fluctuations to maintain homeostasis^65,66^. The altered ambient atmosphere containing cement particulate matter has a profound influence on the biochemical, morphological, and physiological responses of *MD* plants. It is essential to recognise the responses of leaves to different pollutants emitted by different industries^67^, particularly the cement industry. Leaves are fully exposed, abundant, and prominent prime receptors of cement dust and exhibit apparent symptoms of exposure to environmental hazards, such as changes in morphometric features and leaf pigment levels. ROS increases as a result of increased leaf temperature and ultraviolet light blockage resulting from cement dust deposition, and this may lead to the oxidation of cellular components and affect organelle integrity^68^. Toxic pollutants increase oxidative damage by causing H_2_O_2_ accumulation and lipid peroxidation^69^. The overproduction of ROS is a normal event in the oxidative metabolism of plants but their generation is further boosted by the persistent nature of abiotic stress factors (such as cement dust) in a hostile environment. To counteract the environmental effluents, plants develop complex systems of overlapping antioxidants, biometabolites, and enzymes, which work side by side to prevent cellular damage^70^. We found lower ascorbic acid concentrations in leaves of *MD* plants exposed to cement dust, which suggested that *MD* plants were unable to detoxify the accumulated ROS quickly and that the damage to plant tissues was more extensive. This may be attributed to the cement particulate matters that settled on the leaf surfaces that have negative effects on ascorbic acid production. The primary negative effect of higher H_2_O_2_ and MDA levels is the degradation of biochemical constituents. Sugar is a source of energy for all living beings, and its reduction might severely affect energy production in *MD* plant cells exposed to cement dust. Furthermore, a decrease in leaf thickness may result from the cell-damaging effects of cement pollutants. The target components of the plant that are vulnerable to stresses are photosynthetic components, and they have been readily recognised by changes in pigment contents, reduced photosynthetic rates, damaged chloroplast structures, and restricted enzymatic activities or electron transport^70,71^. The sensing of abiotic stresses initiates several complex signalling pathways in plants that trigger alterations in the levels of various leaf pigments^71^. Photosynthetic pigments work under photosystem-II and photosystem-I in plant leaves^72^, in which photons are caught from blue and red lights and carbon dioxide in gaseous exchange processes through stomatal opening to produce oxygen and carbohydrates, such as sugars and amino acids. Coating leaves with cement dust blocks the entrance of sunlight, which might lead to a decline in photosynthetic pigments and subsequently photosynthetic efficiency. Biochemical contents are important for plants to increase their vigour^73^ and are involved in the sensitive core reaction to stress. Cement dust deposition on leaves causes declines in the sugar and amino acid contents of leaves, which leads to the plant’s reduced capability to perform physiological functions and sustain health. After long-term cement dust exposure, fruit toxicity and the effects of consumption on humans and animals should be investigated.

## Conclusions

In conclusion, this work showed the quantitative and qualitative responses of *MD* leaves exposed to different cement dust concentrations. Cement dust exposure has negative effects on *MD* plants. We demonstrated that chronic exposure to cement dust induces abiotic stress, generating unwanted ROS, damaging plant pigments, depleting plant biometabolites, and impairing the histological characteristics of leaves and buds on *MD* plants, resulting in their poor growth and development. Exposure to cement dust triggers a decrease in photosynthetic pigments in *MD,* and it might seriously affect plant photosynthesis. Further studies are needed to address the effects of cement residues in fruits and any health hazards resulting from their consumption.

## Supplementary information

Supplementary Information.
