# Supplementary material for: Chronic cement dust load induce novel damages in foliage and buds of Malus domestica
Source: Sci Rep. 2020 Jul 22;10:12186. doi: 10.1038/s41598-020-68902-6 (PMC7376120; doi:10.1038/s41598-020-68902-6)
Supplement: Supplementary file 1 — Supplementary Information. [file 41598_2020_68902_MOESM1_ESM.pdf]

Supplementary Information

Title: Chronic cement dust load induce novel damages in foliage and buds of *Malus domestica*

Kamran Shah[1, †], Na An[1, †], Wenchun Ma[1], Gulshan Ara[2], Kawsar Ali[3], Svetlana Kamanova[4], Xiya Zuo[1], Mingyu Han[1, *], Xiaolin Ren[1, *], Libo Xing[1, *]

[1]College of Horticulture, Northwest Agriculture and Forestry University, Yangling, 712100 Shaanxi, China.
[2]Institute of Biotechnology and Genetic Engineering, The University of Agriculture, Peshawar, Pakistan.
[3]Agronomy Department, Abdul Wali Khan University Mardan, Pakistan.
[4]College of Food Science, Northwest Agriculture and Forestry University, Yangling, 712100 Shaanxi, China.

Correspondence
Mingyu Han
hanmy@nwsuaf.edu.cn
Xiaolin Ren
renxl@nwsuaf.edu.cn
Libo Xing
libo_xing@nwsuaf.edu.cn
Telephone: +86-15129227289
† These authors contributed equally to this work.

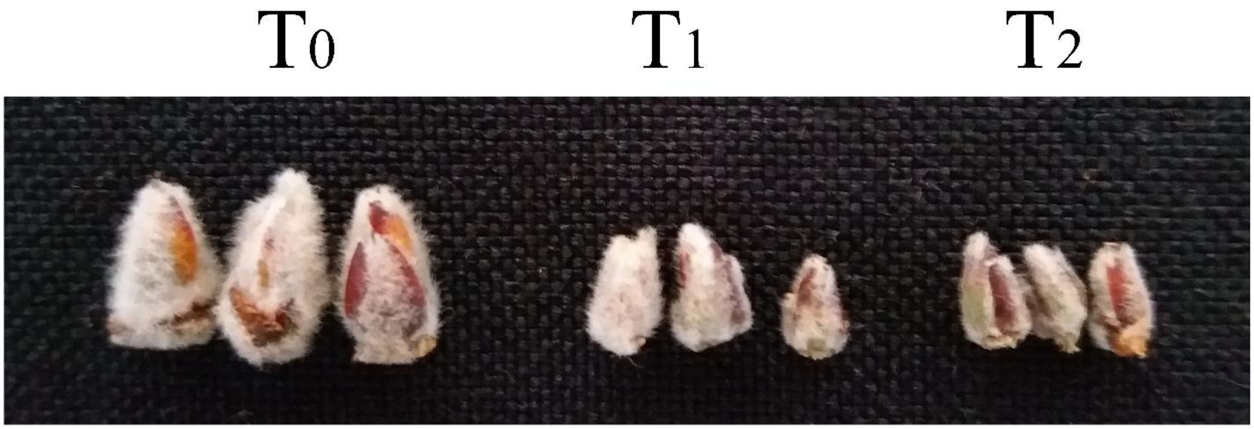

Figure S1. Buds phenotype of *Malus domestica* as affected by T_0 (0 g/plant - control), T_1 (10 g/plant), and T_2 (20 g/plant).
